# Graphene-Modified Composites and Electrodes and Their Potential Applications in the Electro-Fenton Process

**DOI:** 10.3390/ma13102254

**Published:** 2020-05-14

**Authors:** Tian Yu, Carmel B. Breslin

**Affiliations:** Department of Chemistry, Maynooth University, Maynooth, Co. Kildare, Ireland; Tian.Yu.2020@mumail.ie

**Keywords:** electro-Fenton, graphene, oxygen reduction reaction, advanced oxidation, hydrogen peroxide

## Abstract

In recent years, graphene-based materials have been identified as an emerging and promising new material in electro-Fenton, with the potential to form highly efficient metal-free catalysts that can be employed in the removal of contaminants from water, conserving precious water resources. In this review, the recent applications of graphene-based materials in electro-Fenton are described and discussed. Initially, homogenous and heterogenous electro-Fenton methods are briefly introduced, highlighting the importance of the generation of H_2_O_2_ from the two-electron reduction of dissolved oxygen and its catalysed decomposition to produce reactive and oxidising hydroxy radicals. Next, the promising applications of graphene-based electrodes in promoting this two-electron oxygen reduction reaction are considered and this is followed by an account of the various graphene-based materials that have been used successfully to give highly efficient graphene-based cathodes in electro-Fenton. In particular, graphene-based composites that have been combined with other carbonaceous materials, doped with nitrogen, formed as highly porous aerogels, three-dimensional materials and porous gas diffusion electrodes, used as supports for iron oxides and functionalised with ferrocene and employed in the more effective heterogeneous electro-Fenton, are all reviewed. It is perfectly clear that graphene-based materials have the potential to degrade and mineralise dyes, pharmaceutical compounds, antibiotics, phenolic compounds and show tremendous potential in electro-Fenton and other advanced oxidation processes.

## 1. Introduction

As the quality of water continues to decrease, there has been an ever-increasing interest in advanced oxidation processes (AOPs) that are capable of mineralising organic pollutants to CO_2_, H_2_O and inorganic ions, or at least to harmless products [1]. These organic pollutants, which include pesticides, herbicides, dye molecules, phenolic compounds, antibiotics, pharmaceuticals and surfactants, are normally very difficult to degrade [2,3]. Conventional water treatment plants are not always capable of removing these emerging contaminants. Although they are present in water at relatively low concentrations, their presence and ability to produce even more harmful metabolites have led to the development of AOPs. These processes range from UV irradiation and ozonation to electrochemical oxidation [1,4,5]. The electro-Fenton (E-Fenton) process has been identified as a particularly attractive technology, as it is clean and can be used to generate reasonably high concentrations of hydroxy radicals, OH^•^, that can be employed in the oxidation, degradation and mineralisation of various organic compounds and is considered to be one of the more promising and emerging AOPs [1,6,7]. 

The main principle of the E-Fenton process is summarised in Equation (1), where the oxidation of Fe^2+^ to Fe^3+^ facilitates the conversion of H_2_O_2_ to the highly oxidising OH^•^, with a standard reduction potential of 2.56 V vs. SCE (saturated calomel electrode). Consequently, these OH^•^ radicals can be employed to mineralise a large number of organic contaminants. The sustained production of OH^•^ requires both Fe^2+^ and H_2_O_2_. The Fe^2+^ ions can be regenerated through the reduction of Fe^3+^ at the cathode (Equation (2)), provided the Fe^3+^ ions do not form hydroxide precipitates in the solution phase, to give a near-continuous supply of Fe^2+^. The H_2_O_2_ is produced by the two-electron reduction of dissolved oxygen; see Equation (3).

Fe^2+^ + H_2_O_2_ + H^+^ → Fe^3+^ + OH^•^ + H_2_O (1)

Fe^3+^ + e^−^ → Fe^2+^ (2)

O_2_ + 2H^+^ + 2e^−^ → H_2_O_2_ (3)

However, the classical homogeneous E-Fenton process suffers from a number of limitations. The generation of secondary sludge, as ferric and ferrous ions in the treated wastewater, gives hydroxide precipitates and these must be removed, adding cost and reducing the overall efficiency. To limit the formation of solid Fe(OH)_2_ and Fe(OH)_3_, the system must be operated under stringent pH control as these hydroxide species are only soluble at pH values lower than about 4.0. Therefore, the pH of water samples or effluents must be acidified and brought to an acidic pH value of approximately 2.0 to 3.0. Moreover, the OH^•^ radicals are not continuously generated and require a supply of pure oxygen. 

**Figure 1:**
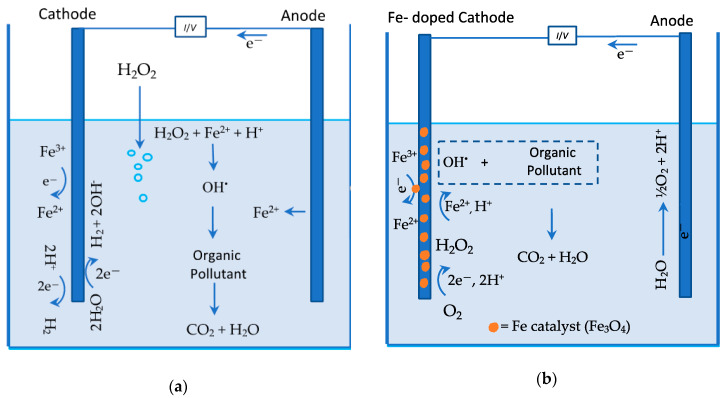
Schematic of (**a**) homogeneous and (**b**) heterogeneous electro-Fenton (E-Fenton).

In more recent years, heterogeneous E-Fenton has emerged as a solution to the issues with iron hydroxide precipitation [8,9,10]. In this case, iron catalysts are incorporated as solids, usually oxides, such as Fe_3_O_4_, into a suitable electrode material. In Figure 1, a schematic is provided, illustrating homogenous and heterogeneous E-Fenton. As shown in Figure 1a for homogeneous E-Fenton, Fe^2+^ is generated from a sacrificial anode and reacts with the H_2_O_2_ in the bulk solution. An iron salt can also be added to the cell to facilitate this reaction. In contrast, the aim in heterogeneous E-Fenton (Figure 1b) is to maintain the Fe^2+^/Fe^3+^ couple in the solid state [11] and, provided the cathode promotes the two-electron reduction reaction to give H_2_O_2_, these coupled reactions can be sustained. 

**Figure 2:**
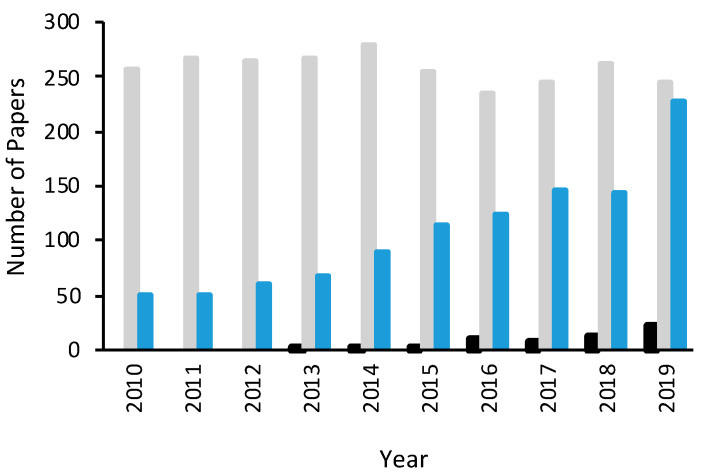
Number of publications shown as a function of the year of publication, taken from Scopus, for Fenton (grey), E-Fenton (blue) and E-Fenton coupled with graphene-based materials (black).

Although heterogeneous E-Fenton can be employed over a wide pH range, issues still remain with the sluggish reduction of Fe^3+^ to Fe^2+^ within the iron-containing catalysts and long-term instability of the catalysts. Furthermore, high and sustainable amounts of H_2_O_2_ are required to provide efficient levels of OH^•^ and this can be difficult as the two-electron oxygen reduction reaction is often complicated by the competing four-electron transfer reaction. Therefore, it is no surprise that considerable effort has been devoted to the design and production of cathode materials that facilitate the formation of high yields of H_2_O_2_. Metals such as Au, Pt, Pd and Ru [12,13] are effective catalysts for the production of H_2_O_2_, with relatively low overpotentials and very good conductivity. However, these are not cost-effective. Recently, carbon-based electrodes and, in particular, graphene-based materials are beginning to be employed in E-Fenton cells and in E-Fenton technologies. This developing interest in the use of graphene-based materials in E-Fenton can be seen clearly in Figure 2, where the number of publications is shown as a function of the year of publication for Fenton, which covers the classical Fenton reagents, E-Fenton, and graphene-based materials coupled with E-Fenton. The publications assigned to Fenton include E-Fenton and this comparison highlights the rise in the popularity of E-Fenton over recent years. It is also very evident from this analysis that graphene-based materials are being increasingly considered as electrodes in E-Fenton cells and are likely to make a more significant impact in the near future.

In this review, graphene-modified electrodes and composites and their applications in E-Fenton are reviewed and discussed. There are a number of review papers devoted to AOPs and E-Fenton technologies [14,15,16,17], for example, Brillas and Martinez-Huitle [15] have reviewed various electrochemical treatments, including OH^•^ radicals as oxidants; Nidheesh et al. [18] have reviewed various electrochemical advanced oxidation processes for the removal of dye molecules, while Bechelany and co-workers [19] have considered a number of carbonaceous materials for energy and environmental applications, such as E-Fenton. Likewise, there are some excellent reviews published on graphene/rGO and its applications [20,21]. Nag et al. [20] have described the applications of graphene/rGO in sensors, Chang et al. [21] have reviewed the use of graphene-based materials as anodes in batteries, while the applications of graphene-based composites to electrocatalysis [22], energy storage [23] and flexible electronics [24] have all been described and reviewed. However, to the best of our knowledge, there is only one mini-review that considers graphene-based cathodes in E-Fenton [25]. In this present review, we consider a more extensive variety of graphene-based composites and electrodes and discuss their emerging applications in E-Fenton. The methods employed in forming these composites and their subsequent performance in catalysing the selective two-electron oxygen reduction reaction and the removal of a number of micropollutants and organic contaminants in E-Fenton are reviewed and discussed.

## 2. Oxygen Reduction Reaction and Graphene-Based Electrodes

The oxygen reduction reaction is important in several applications [26,27] and occurs by two different pathways, depending on the solution pH and cathode material. The two-electron reduction reaction is illustrated in Equation (3), while the competing four-electron reduction is given in Equation (4).

O_2_ + 4H^+^ + 4e^−^ → 2H_2_O (4)

As high yields of H_2_O_2_ are needed to provide the essential OH^•^ species, the two-electron reduction reaction is the preferred reaction in the E-Fenton cell and therefore new catalysts are required to give high selectivity for the two-electron over the four-electron oxygen reduction reaction. Not only is this reaction highly relevant to E-Fenton, but H_2_O_2_ is used in a number of other applications and it is currently formed using an energy-demanding anthraquinone oxidation process [28]. Consequently, it is no surprise that, in the past decade, considerable attention has been devoted to designing new materials that can be employed to generate H_2_O_2_ through the two-electron reduction of dissolved oxygen [29,30]. Despite this considerable interest, the origin of the selectivity is still poorly understood. The four-electron transfer reaction is favoured by thermodynamics, which may indicate that selectivity for the two-electron transfer reaction originates in kinetics [31]. In addition to selectivity issues, there are a number of parasitic reactions that can occur at the cathode or in the bulk solution [29]. The reduction of H_2_O_2_ to H_2_O may take place at the cathode–solution interface [32] (Equation (5)), or it may give O_2_ and H_2_O through a disproportionation reaction [33] (Equation (6)), or be oxidised at the anode in the cell, or through the generation of the HO_2_^•^ intermediate (Equation (7)) [34]. It is generally accepted that the highest yield of H_2_O_2_ is achieved at pH values between 2.0 and 3.0 [35], with more acidic conditions favouring the reduction of H^+^, while the lack of protons with increasing pH reduces the rate of the reaction.

H_2_O_2_ + 2e^−^ + 2H^+^ → 2H_2_O (5)

H_2_O_2_ → O_2_ + 2H_2_O (6)

H_2_O_2_ → HO_2_^•^ + H^+^ + e^−^ → O_2_ + 2H^+^ + 2e^−^ (7)

In an attempt to overcome these complex issues, various carbon-based materials have been considered, as these are generally cost effective and facilitate the two-electron reduction of oxygen. In particular, carbon in different forms, such as amorphous carbon, glassy carbon, graphite, fullerenes and carbon nanotubes have all been evaluated [29]. However, research is increasingly focused on the graphene family, as it presents a genuine alternative, with high surface area, moderate to good conductivity, excellent stability and its properties can be tailored using heteroatom doping and it can be easily combined with other materials [36,37,38,39]. For example, Yang et al. [40] employed a graphite felt electrode modified with electrochemically generated exfoliated graphene and carbon black and reported a H_2_O_2_ production rate of 7.7 mg h^−1^ cm^−2^.

Graphene is a two-dimensional material with *sp*^2^-hybridised carbon atoms arranged in a two-dimensional honeycomb monolayer to produce a one-atom-thick sheet. Graphene can be produced using a variety of both top-down and bottom-up approaches, including exfoliation, sonication, ball milling, chemical vapour deposition and epitaxial growth [41]. However, the production of pristine graphene with minimum defects is still challenging and while mechanical cleavage of graphite results in high quality graphene flakes, the yield is low, making the mass production of graphene demanding and time consuming [41]. One of the more common routes for the production of graphene-based materials is the formation of graphene oxide (GO) followed by its reduction to reduced GO (rGO) [42,43,44]. GO is typically synthesised by oxidising graphite in a mixture of NaNO_3_, KMnO_4_ and H_2_SO_4_, which is widely known as the modified Hummers method [42,43,44,45,46]. Once the graphite is oxidised, the interlayer spacing increases and the resulting expanded interlayer, combined with ultrasonication, allows for liquid-phase exfoliation in order to produce the GO sheets.

This oxidation process introduces a number of negatively charged oxygen-containing groups, such as hydroxy and carboxy groups, to the GO sheets and as a result, the GO sheets have good hydrophilic properties. GO exhibits good stability as a colloidal solution and this is very useful for solution processing approaches. The GO sheets can be subsequently reduced to give rGO, which has a much higher conductivity, and it is this member of the graphene family that is most suitable for electrochemical applications, such as the reduction of dissolved oxygen to produce H_2_O_2_ in E-Fenton applications. The reduction methods employed are generally thermal or chemical reduction processes [47]. The thermal reduction involves heating the GO to temperatures in the vicinity of 400 to 1100 °C, where most of the oxygen-containing groups are transformed to gaseous CO or CO_2_, giving a reduction of GO and the formation of rGO. Various reducing agents, such as borohydride, hydrazine or hydrogen iodide can be employed at room temperature or with mild heating to give rGO. However, some of these chemicals have environmental concerns and they can introduce impurities to the carbon matrix. These health and environmental issues can be overcome by using ascorbic acid as the reducing agent. This has been successfully employed to reduce GO and the resulting rGO was shown to have good conductivity [48]. The electrochemical reduction of GO is another very effective and simple route to obtain rGO [49,50,51]. For example, Guo et al. [50] used a potential of −1.5 V to reduce GO, giving a green and fast process with no evidence of contamination of the rGO sheets. However, the rGO produced from all these approaches contains some oxygen-containing functional groups. 

Different approaches have been used in forming graphene-based cathodes in an attempt to give enhanced and selective production of H_2_O_2_. These approaches include various electrophoretic and electrodeposition routines to generate graphene-based cathodes, graphene-based materials deposited from slurries or suspensions at graphite or carbon felt electrodes which act as supports, graphene-based inks, porous graphene-based aerogels, three-dimensional graphene-modified electrodes, heteroatom doped graphene composites, graphene composites combined with iron oxides and graphene-based diffusion electrodes. These are now described and discussed in the following sections.

### 2.1. Graphene Modified Carbon/Graphite Felt Electrodes and Other Supports 

One of the most common approaches is to use carbon or graphite felt as a support for GO or rGO as these three-dimensional felt cathodes have low cost, high surface area, excellent conductivity and high porosity. The exfoliated GO/rGO can be deposited at the carbon or graphite electrodes using electrophoretic deposition [52], coated from a liquid solution phase with different additives, such as polytetrafluoretyhylene (PTFE) and carbon black to form a slurry, followed by a heating or an annealing process of the electrode [39,53,54]. Various additives have been employed in formulating these graphene-containing suspensions or slurries. In several papers polytetrafluroethylene (PTFE), a synthetic fluropolymer with good adhesion and lubricating properties, has been used successfully [55,56]. Spin coating has also been employed to deposit GO from slurries [57], while in some cases, the treated carbon or graphite felt is immersed in the rGO containing suspension or slurry [4], or dip-coated [58,59]. The solution or slurry coating approaches enable the addition of various additives that have the potential to enhance the removal of micropollutants using E-Fenton. However, the addition of binders, such as PTFE, can reduce the electric conductivity and increase the impedance of the final composite [60], while hindering ion permeability at the electrode-solution interface [61]. The electrophoretic deposition routines can be easily coupled with the electrochemical reduction of GO to form rGO without the need for reducing agents and binders that are toxic in many cases. Interestingly, in a comparison of the reduction of GO to rGO using a constant potential reduction, chemical reduction and thermal reduction, it was concluded that the electrochemical reduction was the best option, in terms of simplicity, cost, ecology and performance in the mineralisation of an azo dye [62]. 

Indeed, these electrochemical approaches, employing combinations of electrochemical exfoliation and electrophoretic deposition, followed by the reduction of GO to rGO, have been used to coat carbon felt electrodes [52], while in some cases the electrochemically exfoliated GO is combined with carbon black before being deposited at the felt electrodes [3,40]. These graphene-modified electrodes have been shown to faciliate the oxygen reduction reaction, producing H_2_O_2_ with low energy consumptions of 9.7 kWh kg^−1^ [39] and 3.08 kWh kg^−1^ [3] and have been employed successfully to remove acid orange [52], sulfadiazine [3] and imatinib [55]. 

On the other hand, graphene-containing slurries can be readily formed using exfoliated or electrochemically exfoliated GO and these have been combined with quinones (AQ), which have the ability to generate H_2_O_2_ (Equations (8) and (9)). For example, Gao et al. [53] used GO, PTFE and anthraquinone sulfonate to modify carbon felt to fabricate a hybrid electrode. The catalytic oxygen reduction reaction was greatly enhanced in the presence of the quinone, resulting in the efficient removal of Rhodamine B. The authors proposed a mechanism whereby a semi-quinone (s-Q) anion radical is formed at the cathode (Equation (10)). This is followed by a catalysed reduction of dissolved oxygen to generate the oxygen radical anion (Equation (11)), which then combines with protons to generate H_2_O_2_; see Equation (12).

AQ + 2H^+^ + 2e^−^ → AQH_2_  *E*^0^ = 0.43 V vs. SCE (8)

AQH_2_ + O_2_ → AQ + H_2_O_2_ (9)

AQ + e^−^ → *s*-Q^•−^ (10)

O_2_ + *s*-Q^•−^ → O_2_^•−^ + *s*-Q (11)

2O_2_^•−^ + 2H^+^ → H_2_O_2_ + O_2_ (12)

It is well established that a number of oxygen-containing functional groups, such as epoxides (C–O–C), hydroxy (OH), carboxylic (COOH) and carbonyl groups (C=O) are present on GO nanosheets [63]. However, other oxygen-containing groups, such as ketones and quinones have been detected. Aliyev et al. [64], using a combination of surface analytical techniques, clearly identified quinone groups on GO layers, and these may also contribute to the generation of H_2_O_2_ when GO/rGO is employed as the cathode material. Indeed, Nambi and co-workers [65] attributed some of the enhanced production of H_2_O_2_ and the degradation efficiency of E-Fenton to quinone functional groups on electrochemically exfoliated rGO.

Graphene oxide can be easily functionalised with ferrocene [66,67], and ferrocene-functionalised rGO has been deposited at graphite felt electrodes. For example, Nambi and co-workers [58] designed a cathode by fabricating ferrocene-functionalised rGO on graphite felt electrodes and studied its heterogeneous E-Fenton reaction for the degradation of ciprofloxacin at neutral pH conditions. The removal rate of ciprofloxacin was computed as 0.035 min^−1^ for the ferrocene-modified rGO electrode, significantly higher than the value of 0.004 min^−1^ obtained for the unmodified graphite felt and also higher than 0.010 min^−1^, which was the rate constant observed with the reduced rGO-modified felt electrode. The authors concluded that the rGO and ferrocene participated in sequential steps, with rGO facilitating the production of H_2_O_2_, while the Fe^2+^ centre in ferrocene catalysed the decomposition of H_2_O_2_ to form OH^•^ and Fe^3+^-centred ferricenium. The same group studied the ferrocene-functionalised rGO felt electrode as an E-Fenton catalyst using rotating disc voltammetry for the removal of ciprofloxacin and carbamazepine [59]. Using rotating disc voltammetry, which gives improved mass transfer, a continuous supply of reactive oxygen species was achieved without aeration of the solution, to give OH^•^ concentrations of 644 µM, 264 µM and 163 µM at pH values of 3.0, 7.0 and 9.0, respectively, facilitating the removal of contaminants over a wide pH range. 

Using another approach, Mi et al. [4] combined rGO with WO_3_ and Ce and deposited the hybrid composite, rGO/Ce/WO_3_, on carbon felt. This composite was employed in E-Fenton to remove ciprofloxacin with complete degradation within 1 h, and a mineralisation degree of 98.55% within 8 h. It was suggested that the Ce^3+^ catalysed the decomposition of H_2_O_2_ to give the OH^•^ radical species (Equation (13)), while superoxide was generated from the reaction between Ce^3+^ and dissolved O_2_ (Equation (14)) and between W^5+^ and O_2_ (Equation (15)). The Ce^4+^ was recycled to Ce^3+^ through a simple reduction step (Equation (16)), while Fe^2+^ ions added to the E-Fenton cell also facilitated the reduction of Ce^4+^ (Equation (17)).

Ce^3+^ + H_2_O_2_ + H^+^ → Ce^4+^ + OH^•^ + H_2_O (13)

Ce^3+^ + O_2_ → Ce^4+^ + O_2_^−^^•^ (14)

W^5+^ + O_2_ → W^6+^ + O_2_^−^^•^ (15)

Ce^4+^ + e^−^ → Ce^3+^ (16)

Ce^4+^ + Fe^2+^ → Ce^3+^ + Fe^3+^ (17)

Graphene/GO has also been combined with conducting polymers and used in E-Fenton. In a recent study, a simple electropolymerisation method was employed to deposit poly (3,4-ethylenedioxythiophene) (PEDOT), sodium polystyrene sulfonate (NaPSS) and GO on graphite felt electrodes [68]. This GO/PEDOT:NaPSS showed much greater rates of H_2_O_2_ production and greater efficiency in the degradation of methylene blue in heterogeneous E-Fenton compared with the GO-free PEDOT:NaPSS. This was attributed to a synergistic effect between PEDOT and GO, promoting higher electron transfer rates.

In addition to electrophoretic deposition and coating from graphene-containing slurries, there has been considerable interest in graphene-based inks that can be painted or printed onto substrates [69,70]. This approach has been employed to produce graphene-based cathodes in E-Fenton [6,71]. Conducting graphene-based inks have been formed in ethanol and water mixtures using Nafion as a binder and dispersant, and the resulting ink coated onto carbon cloth [71] and carbon fibres [6]. These ink-coated cathodes have been shown to give rise to a near doubling of the amount of H_2_O_2_ generated and a three-fold increase in the rate of phenol degradation. A graphene-based paste cathode has also been employed to degrade a pharmaceutical product [72]. The paste was formed by mixing rGO with graphite powder and paraffin, which served as the binding agent. Divyapriya et al. [73] used another approach, whereby a liquid crystal display glass was utilised as a supporting matrix for the deposition of a thin graphene-modified electrode without the need for binders or linkers. GO was drop casted on the glass and electrochemically reduced to rGO and then used for the oxidation and degradation of ciprofloxacin. The authors showed that the drop cast electrode exhibited good stability. Graphene oxide has also been deposited on stainless steel to create a membrane for the removal of paracetamol [74], and an electrode to oxidise and remove arsenic [5], while a PTFE membrane was modified with graphene and used as a catalytic membrane to both concentrate and oxidise an antibiotic [75].

In most of these approaches, the graphene-modified electrode is compared to the conducting carbon or graphite felt or carbon cloth substrate electrodes and the addition of rGO clearly enhances the rate of H_2_O_2_ generation to give a more efficient removal of the contaminants. Interestingly, it was shown by Wang et al. [57], who employed polyvinylidene difluoride to fabricate both carbon nanotube (CNT) modified felt and graphene-modified felt electrodes, that the graphene-modified carbon felt was superior in the degradation of an azo dye molecule, reaching degradation rates of 70.1%, compared to the lower rates of 55.3% evident with the CNT-modified electrode. While both modifications enhanced the azo dye degradation rate compared to the untreated carbon felt electrode, the graphene-modified electrode produced a higher quantity of H_2_O_2_. This suggests that the graphene-modified electrodes are not only more superior than the conducing graphite or carbon substrates used in their fabrication, but they may also be more efficient in producing H_2_O_2_ than carbon nanotubes. The impressive performance of the graphene-modified carbon/graphite felt electrodes is clearly illustrated in Table 1. For comparative purposes, some very recently optimised and high-performing graphite-based electrodes are included. Although the amount of H_2_O_2_ generated is expressed differently, sometimes as rates and in other cases as mass per volume, it is evident that a graphene-based system can be employed to give the more efficient generation of H_2_O_2_ with relatively high rate constants for the removal of a variety of contaminants.

### 2.2. Porous Graphene Electrodes

Porous graphene-based composites that can be employed as aerogels, foams and as gas diffusion electrodes are interesting and these new materials are beginning to emerge in E-Fenton. 

#### 2.2.1. Graphene Aerogels

**Figure 3:**
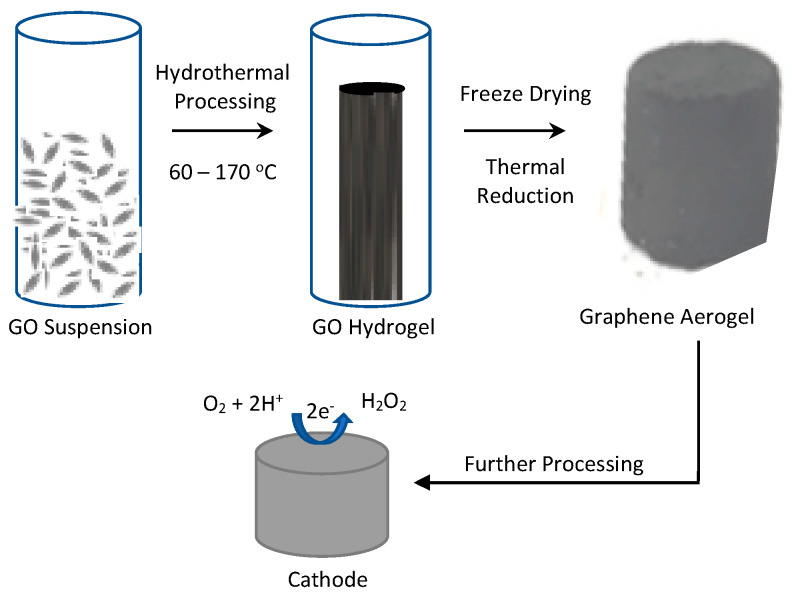
Schematic illustrating the formation of a graphene-based aerogel.

Aerogels are three-dimensional highly porous materials and graphene-based aerogels exhibit interesting properties of high thermal stability, surface area and electrical conductivity [78]. They are readily constructed by the self-assembly of rGO to form a three-dimensional macroporous architecture [79,80], through template-guided approaches, solvothermal sol–gel reactions, or patterning technologies [81]. A schematic illustrating the formation of an aerogel from graphene-containing dispersions using the hydrothermal route is provided in Figure 3. The synthesised aerogel can be further processed, formed into discs and used as cathodes. These emerging materials are finding applications in fuel cells [82], environmental remediation [81,83], batteries [84] and, more recently, as electrodes in E-Fenton [85,86,87]. In particular, the aggregation and restacking of the rGO layers are minimised by the three-dimensional structure inside the bulk aerogel, giving good stability, while the surface layers are available to the electrolyte ions and target pollutant molecules and can be tailored for high pollutant adsorption. In addition, these materials are promising in heterogeneous E-Fenton, where zero-valent iron nanocrystals/nanoparticles or Fe_3_O_4_ nanoparticles can be embedded and protected within the aerogel [85]. A number of studies has been reported where rGO aerogels have been assembled and successfully used in E-Fenton for the removal of various contaminants [85,87,88,89].

For example, Wen et al. [87] prepared three-dimensional macroporous rGO aerogels through the in situ assembly of rGO sheets. This electrode was employed as a cathode in E-Fenton to degrade the complex formed between ethylenediaminetetraacetic acid (EDTA) and Ni^2+^, EDTA-Ni. Using surface analytical techniques and adsorption measurements, the authors confirmed that the three-dimensional structure possessed homogenous macropores with a high surface area of 280.15 m^2^ g^−1^. The cathode showed enhanced electrocatalytic activity, leading to the efficient generation of H_2_O_2_ and regeneration of Fe^2+^. Likewise, Nazhif Mohd Nohan et al. [89] used a one-pot hydrothermal procedure to synthesise composites comprising CNTs and rGO aerogels. Studies revealed that the CNTs improved the surface area, pore volume and conductivity. 

Graphene-based aerogels containing trapped Fe_2_O_3_ nanoparticles have also been successfully fabricated and employed in the heterogenous E-Fenton system for the degradation of Rhodamine B [88]. The addition of the Fe_2_O_3_ nanoparticles enhanced the degradation rate and this was attributed to the generation of H_2_O_2_ within the aerogel and the good diffusion and electrosorption of Rhodamine B within the aerogel to give high concentrations of the accumulated contaminant. The H_2_O_2_ was decomposed in the presence of Fe_2_O_3_ to give oxidising OH^•^ with little quenching, as high concentrations of the pollutant were available. Chemical oxygen demand elimination rates up to 82% were observed, while a degradation rate of 99% was reached after 30 min. Low iron leaching rates were observed even in acidic media and there was no significant loss in the catalytic activity over six cycles. In a separate study, a carbon aerogel with rGO, CNT and iron oxide nanoparticles, Fe_3_O_4_, was formed successfully using a sol–gel process and then employed in an E-Fenton cell as a cathode in the degradation of methyl blue [85]. The good removal efficiency, reaching 99% after a 60 min period, was attributed to the high adsorption ability of the graphene-based composite and the added strength introduced by the CNTs.

#### 2.2.2. Three-Dimensional Graphene-Based Electrodes

In terms of three-dimensional graphene-based materials, there is some evidence that three-dimensional graphene-modified foams may be suitable in E-Fenton. In a comparison of graphene-based monolayer, multilayer and three-dimensional graphene-based foams as cathode materials in E-Fenton for wastewater treatment and the mineralisation of phenol, it was found that the three-dimensional foam exhibited the highest H_2_O_2_ electrogeneration yield, degradation and mineralisation rates [90]. The superiority of the three-dimensional graphene was attributed to its low interfacial charge transfer resistance, high surface area and porous structure. Interestingly, Roman et al. [28] have shown that a selective two-electron oxygen reduction reaction can be achieved with a selectivity of 94 ± 2% using three-dimensional out-of-plane graphene edge sites. This was achieved by tuning the synthesis conditions to control the size and density of the out-of-plane graphene flakes and edges to provide a three-dimensional fuzzy graphene. In addition to the high selectivity, the onset potential was measured as 0.79 V vs. reversible hydrogen electrode (RHE).

#### 2.2.3. Three-Dimensional Graphene-Modified Electrodes

**Figure 4:**
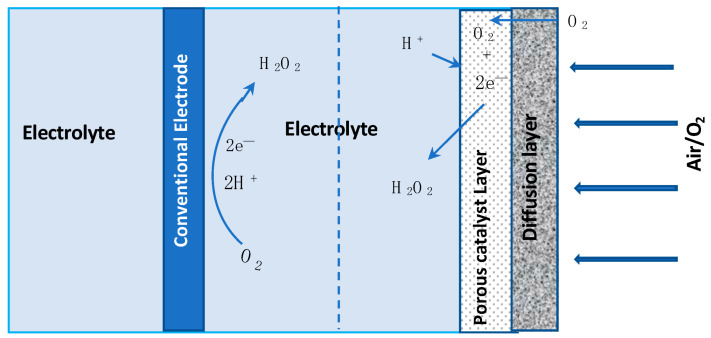
Schematic diagram of conventional solid-state electrode and porous gas diffusion electrode where the solid catalyst layer coexists with gas and liquid phases.

Gas diffusion electrodes (GDE) are being used increasingly in fuel cells [91], batteries [92,93] and in the generation of H_2_O_2_ [94,95]. They are attractive in E-Fenton, as the solubility of dissolved oxygen is very low in water and, by using these GDE electrodes, the oxygen in air could be employed instead. The GDE electrode consists of a gas diffusion layer and the solid catalyst in contact with the aqueous phase, consisting of solid, liquid and gaseous phases. Normally, the catalyst layer is porous and the aerogels described in Section 2.2.1 are finding applications as catalyst layers, facilitating interactions between the liquid and gas phase. A schematic diagram illustrating the difference between the oxygen reduction reaction at a conventional cathode and at a gas diffusion electrode is presented in Figure 4. As illustrated in this schematic, the air or gaseous phase is in contact with one side of the catalyst, enabling the diffusion of oxygen through the micropores of the diffusion layer to the catalyst phase, where it reacts with H^+^ from the aqueous phase, while the conducting catalyst provides the electron, facilitating the formation of H_2_O_2_. Recently, this approach is finding applications in E-Fenton [96,97,98]. However, in many of these reports, pure oxygen is pumped to the surface of the gas diffusion cathode, rather than having the typical gas/air, solid and liquid phases, as illustrated in Figure 4.

For example, a graphene–graphite diffusion electrode with high conductivity and electrocatalytic activity was formed for the continuous in situ generation of H_2_O_2_ through the oxygen reduction reaction, for the removal of Rhodamine B [56]. A removal rate of 98% was achieved after a 60 min period, which was higher than that obtained with a graphite-based gas diffusion cathode or graphite sheet cathode. The significant difference between the gas diffusion cathode and sheet cathode was attributed to the concentration of oxygen available for reaction, with the oxygen being supplied directly to the surface of the gas diffusion electrode, which, in turn, accelerated the generation rate of H_2_O_2_. The porous structure of the graphene-based diffusion electrode provided a reaction chamber for the efficient conversion of oxygen to H_2_O_2_. Liu et al. [98] used a graphene-based composite as a gas diffusion electrode for the removal of dimethyl phthalate from aqueous solution. Again, it was found that the H_2_O_2_ production was significantly improved. The apparent rate constant of dimethyl phthalate degradation was computed as 0.0322 min^−1^. The graphene-based diffusion electrode showed excellent recovery and could be reused. However, the degradation rate decreased slightly with increasing use and this was attributed to the blocking of the microspores and channels by iron sludge as the E-Fenton reaction proceeded. In another study, a gas diffusion electrode, consisting of a cylindrical body with a built in air diffuser, was fabricated using carbon cloth treated with PTFE and coated with electrochemically exfoliated rGO for the removal of industrial electronic wastewater [99]. High H_2_O_2_ concentrations of 495 mg L^−1^ were achieved to give a mineralisation rate of 80% over 80 min. Gas diffusion electrodes have also been fabricated by combining rGO with Fe_3_O_4_ [7] and by using boron-doped graphene-based aerogels [100] for the removal of Bisphenol A. The combination of available Fe_3_O_4_ particles adjacent to the electrogenerated H_2_O_2_ facilitated the efficient generation of OH^•^ and the removal of Bisphenol A [7]. A comparison of the aerogels, the porous three dimensional graphene-based composites and gas diffusion electrodes is summarised in Table 2, where it is evident that these materials have high surface areas, with a very good generation of H_2_O_2_, and they have been employed in the removal of several pollutants.

While many studies indicate the superiority of graphene/rGO in fabricating gas diffusion electrodes, there are examples where other carbon-based systems exhibit a somewhat higher catalytic activity for the generation of H_2_O_2_. In a comparative study, tert-butyl-anthraquinone (TBAQ) was used to modify four different carbon materials, carbon aerogel, CNT, carbon black and graphene-doped carbon black to fabricate gas diffusion electrodes for the production of H_2_O_2_ [104]. In this case, it was found that the highest H_2_O_2_ production and current efficiency were achieved with the CNT-gas diffusion electrode modified with 2% TBAQ, giving 2.15 mg h^−1^ cm^−2^ compared to 1.97 mg cm^−2^ h^−1^ of H_2_O_2_ for the corresponding graphene-based material. However, rates higher than 2.15 mg h^−1^ cm^−2^ can be seen in Table 2 for the graphene-based system. On the other hand, gas diffusion electrodes assembled using sulfur-doped carbon nanoparticles have been shown to exhibit superior electrocatalytic activity in an acidic medium for the oxygen reduction reaction [105].

### 2.3. Doping of Graphene-Based Materials

The doping of graphene/rGO, using heteroatoms such as N, B, P and Fe, has been widely studied for the development of catalysts for the oxygen reduction reaction [106,107]. Depending on the preparation conditions employed and the nature of the dopants selected, the mechanism can vary from a two-electron to a four-electron pathway. Much of the research focus for environmental applications is directed at N-doped graphene, as it has shown the more promising results, with many studies indicating that the presence of N enhances the production of H_2_O_2_ [108], while Fe and P co-doping favours the four-electron pathway [109]. Likewise, the addition of P and B to N-doped graphene composites reduces the production of H_2_O_2_ [106]. As N has a higher electronegativity than C, it attracts electrons, generating a partial positive charge on the C atoms, while B and P tend to donate electrons to the C. Both these partial positive and partial negative charges should promote the adsorption of oxygen. However, little is known about the oxygen reduction reaction at N-doped graphene composites or, indeed, the parameters that determine the selectivity of the oxygen reduction reaction at these materials. Several researchers have hypothesised that oxygen adsorbs through a side-on orientation on N-doped carbon [110,111]. However, it has also been shown from theoretical calculations that this side-on adsorption is unlikely and that end-on adsorption is more favourable [112,113]. The three main forms of N in N-doped graphene are pyridinic, pyrrolic and graphitic. Some authors claim that it is the graphitic N sites that facilitate oxygen adsorption and therefore the oxygen reduction reaction [112,114], while others have proposed that it is the pyridinic N that improves the chemisorption of oxygen [115]. It has also been shown that oxygen adsorption occurs on the carbon atoms at edges and these are far removed from the graphitic N atoms, making it difficult to explain how N doping facilitates the oxygen reduction reaction. 

The four-electron transfer pathway is normally observed in alkaline solutions [107], while, in acidic environments, two-electron transfer is the more favoured reaction for carbon-based materials [116]. However, some N-doped or N-treated graphene-based electrodes have been found to catalyse the four-electron reduction in aqueous acid solutions [117,118]. Kurak and Anderson [119] have used a linear Gibbs energy relationship applied to N-doped graphene composites to predict the reversible potential for the formation of oxygen reduction reaction intermediates in acid. These authors concluded that there was no clear pathway for the direct four-electron reduction reaction, suggesting that transition metal impurities may be responsible for the observed direct four-electron pathway. Indeed, it has been suggested that the presence of Cu in N-doped graphene-based materials favours the two-electron pathway, but traces of Ti, Mo, Nb and Ru favour four-electron transfer [120]. Liu et al. [121] showed that by varying the N-doping temperature and microwave heating power, the number of electrons in the oxygen reduction reaction can be controlled, giving a two-electron reaction for the electrochemical degradation of organic contaminants, or the preferred four-electron transfer reaction for applications in metal air batteries. It has also been shown that the selectivity can be controlled by the degree of oxidation, with the more oxidised graphene facilitating a two-electron reduction pathway to give H_2_O_2_, but on reduction with NaBH_4_, the same materials exhibit more selectivity for the four-electron pathway [122]. Kim et al. [123] suggested that the generation of H_2_O_2_ is connected with epoxy or ether groups in the N-doped rGO, while N-doped graphene composites, with abundant quaternary nitrogen species, show the selectivity of the two-electron reduction pathway; however, with pyridinic species, the four-electron pathway is preferred [124]. These studies highlight that, while the two-electron transfer reaction can be achieved with graphene or N-doped graphene-based materials, the selectivity of this reaction depends on how the graphene composite is formed and processed. Moreover, various methods have been employed in doping graphene-based materials with N and this may also influence the mechanism of the oxygen reduction reaction. 

A number of N-doped graphene-based electrodes have been employed successfully in E-Fenton. Li and Zhang [39] employed N-doped graphene and N-doped graphene–graphite felt cathodes in the removal of phenacetin from wastewater, while Fe/N-doped graphene loaded with Fe/Fe_3_C nanoparticles was used for the removal of phenol, methanol, acetone, dichloromethane and diethyl phthalate from real samples [125]. The authors concluded that the Fe/Fe_3_C nanoparticles were encapsulated and protected, preventing the aggregation or leakage of the Fe, while the high-surface-area porous framework, with abundant channels and pores, provided access for the pollutants, while facilitating the formation of H_2_O_2_. A gas diffusion cathode was formed with N-doped graphene composites and CNTs for the removal of dimethyl phthalate [98]. The authors showed that the oxygen reduction reaction was facilitated with N-doping of the graphene composite, with the effective generation of H_2_O_2_ at a relatively low potential of 0.2 V vs. SCE. Yang et al. [126] employed N-doped graphene/graphite felt electrodes for the efficient generation of OH^•^, with a high yield of 6.2 mg h^−1^ cm^−2^ of H_2_O_2_. Su et al. [54] obtained an even higher yield of H_2_O_2_, reaching a value of 8.6 mg h^−1^ cm^−2^, with a selectivity of 78% at neutral pH and a low energy consumption of 9.8 kW h kg^−1^. In another recent study [127], N-doped graphene-based catalysts were found to accelerate the production of H_2_O_2_ and the generation of OH^•^. This N-doped graphene-modified graphite felt electrode was used in the mineralisation of 2,4-dichlorophenoxiacetic acid with an impressive rate of 88% at a pH of 7.0 after 480 min. 

Graphene-based aerogels, where the graphene was doped with N and also co-doped with N and S were compared as cathodes in a heterogeneous E-Fenton cell [128]. The highest pollutant removal rate was observed with the N-doped graphene-based aerogels combined with a carbon–Fe_3_O_4_ catalyst, giving a removal rate of 71%, a mineralisation rate of 51% with good stability and low iron leaching of 0.33 mg L^−1^. Interestingly, it was observed that the N/S-doped graphene facilitated the four-electron oxygen reduction reaction, while it was mainly the two-electron pathway that was observed with the N-doped graphene-based aerogel. While N-doped graphene appears to be superior in the promotion of the two-electron oxygen reduction reaction, Wu et al. [100] have shown that B-doped graphene-based materials may also have applications as cathodes in E-Fenton cells. 

### 2.4. Graphene-Based Materials Combined with CNTs

Graphene/GO can be easily combined with a variety of other components such as metal oxides [129], metal nanoparticles [130] and metal organic frameworks [131]. While the addition of conducting metals or metal oxides can enhance the overall conductivity of the composite, metal-free carbon-based materials have considerable advantages in terms of cost and this has led to the development of graphene and CNT composites. CNTs can be combined with graphene-based materials to further enhance the electrocatalytic activity and conductivity, while acting as a structural support that prevents the graphene-modified sheets or flakes from restacking. These materials are finding applications in the fabrication of cathodes for E-Fenton and in the removal of contaminants. Bridged N-doped graphene and CNT composites, with microscopic three-dimensional structures, have been employed as gas diffusion electrodes and have been shown to enhance the oxygen reduction reaction, compared to graphene-based electrodes, CNT and graphite [98]. Other approaches include binder-free CNTs and graphene-based aerogel electrodes [89], iron oxide-containing CNTs, graphene-based aerogels [85] and graphene combined with CNTs on carbon felt electrodes [57]. In each of these cases, the addition of CNTs has been shown to be beneficial and to enable the more efficient removal of the contaminants.

### 2.5. Graphene-Based Materials Combined with Iron Oxides and Other Metal Oxides

**Figure 5:**
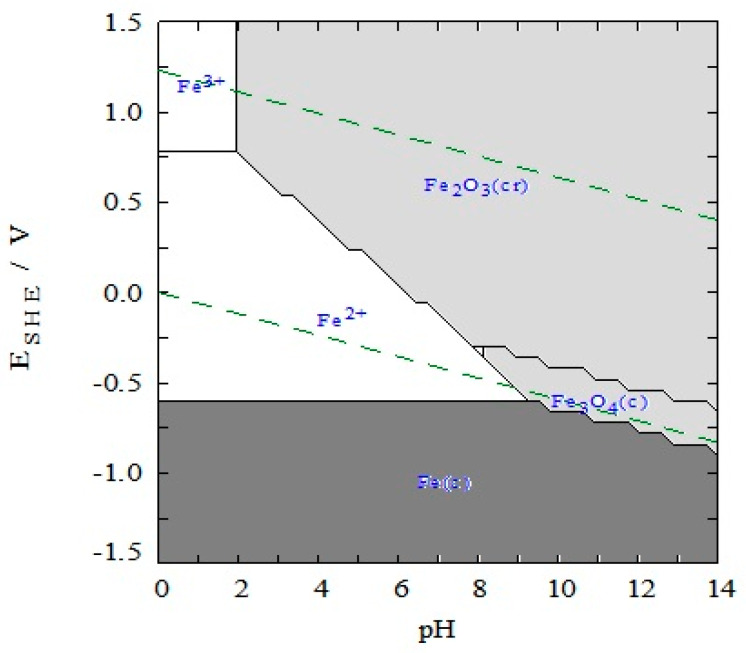
Pourbaix diagram for iron in water (dissolved iron concentration is 1.0 × 10^−5^ M at 298 K). Only Fe, Fe_3_O_4_, Fe_2_O_3_ are considered as the solid products, generated with the MEDUSA software based on the SOLGASWATER algorithm [134].

As detailed earlier, the homogeneous E-Fenton reaction uses the Fe^2+/^Fe^3+^ couple to catalytically decompose H_2_O_2_ into OH^•^, a potent oxidant (and possibly OOH^•^, which is a much weaker oxidising agent). While the Fe^2+^ and Fe^3+^ are soluble in acidic solutions, with any increase in pH, for example through the four-electron reduction at the cathode (Equation (4)), insoluble iron hydroxides begin to form, reducing the concentration of Fe^2+^ ions that are needed for the E-Fenton reaction. This is readily seen from the Pourbaix diagram presented in Figure 5, where the predicted stability phases of the soluble Fe^2+^ and Fe^3+^ are shown as a function of pH. The dashed lines show the pH dependence of the oxygen reduction and hydrogen ion reduction reactions.

Moreover, the reaction rate of Fe^3+^ to Fe^2+^ is approximately 6000 times slower than the oxidation of Fe^2+^ to Fe^3+^, which inhibits efficient recycling between the Fe^2+^ and Fe^3+^ ions [1,132]. In addition, a large mass of iron-containing sludge is formed when the solution is neutralised. These issues can be largely resolved by using heterogenous E-Fenton [11,133]. In this case, solid iron-containing catalysts are immobilised onto a support, which helps to prevent the leaching of iron from the catalyst, minimising sludge formation, giving a wider working pH range and a more efficient conversion of Fe^3+^ to Fe^2+^ in the solid-state catalyst. As shown in Figure 5, at low pH values, leaching and dissolution are more significant, while this dissolution reaction becomes negligible as the pH is increased to neutral or slightly alkaline values. However, the principal aim in heterogeneous E-Fenton is to eliminate the dissolution of iron and the introduction of metal ions into the treated water. In Table 3, iron leaching rates are shown for different graphene-based materials together with the H_2_O_2_ or OH^•^ concentrations formed, where it is seen that relatively low leaching rates can be achieved.

The oxides Fe_2_O_3_, Fe_3_O_4_ and FeOOH, and ferrocene, which all contain the Fe^2+^/Fe^3+^ redox pair, have been employed [138,139,140], while graphene-containing composites have been combined with Fe_3_O_4_ [141,142], FeOOH [102], zero valent iron [143] and ferrocene [58]. In a recent study, Wang et al. [102] fabricated an γ-FeOOH graphene–polyacrylamide-carbonised aerogel (GPCA) for the degradation of sulfamethoxazole. The synthesised γ-FeOOH GPCA cathode had very good conductivity, a high surface area and very good dispersion of the iron component, which facilitated the regeneration of Fe^2+^. This system was employed at a neutral pH to give a total organic carbon removal efficiency of 89%. Zero-valent iron has also been encapsulated within a three-dimensional graphene-based network to give a catalyst for the adsorption and degradation of sulfadiazine [143]. Iron oxide nanoparticles wrapped in graphene-based aerogel with α-Fe_2_O_3_ as the iron source have also been employed in heterogenous E-Fenton [88]. In this case, efficient removal of Rhodamine B was observed, with low iron leaching (<2.3 mg L^−1^) in acidic solutions.

The iron oxide, Fe_3_O_4_, has been used more widely as the iron-containing catalyst [141,144]. For example, a quinone-functionalised graphene-based electrode modified with well-dispersed Fe_3_O_4_ nanoparticles was employed for the continuous electrogeneration of H_2_O_2_ and OH^•^. A degradation efficiency of 98% was observed for the removal of Bisphenol A within 90 min at neutral pH with less than 1% of iron leaching [65]. Akerdi et al. [141] have also employed well-dispersed Fe_3_O_4_ nanoparticles on GO and rGO to enhance the removal of two dyes, methylene blue and acid red. Shen et al. [145] have employed graphene–Fe_3_O_4_ hollow hybrid microspheres, while graphene oxide–Fe_3_O_4_ was employed as a heterogeneous catalyst for the E-Fenton degradation of two antibiotics, chloramphenicol and metronidazole [137]. Fe_3_O_4_ particles have also been utilised with N-doped graphene-based aerogels for the degradation of acetaminophen with a low iron leaching of 0.33 mg L^−1^ in heterogeneous E-Fenton [128]. These iron oxides have also been combined with graphene/CNT aerogel for the degradation of methyl blue [85]. In a more recent study, the efficient decomposition of Bisphenol A was achieved with a gas diffusion electrode and Fe_3_O_4_ particles at N-doped rGO as catalytic particle electrodes. The Fe_3_O_4_/N-rGO also served as the heterogeneous catalyst, resulting in the rapid regeneration of Fe^2+^ and high concentrations of OH^•^ oxidants [7].

There is also increasing interest in finding iron-free or, indeed, metal-free catalysts for heterogeneous E-Fenton. An iron-free rGO/MoS_2_/Ce_0.75_Zr_0.25_O_2_ composite was fabricated and used for the effective removal of ciprofloxacin. The decomposition of H_2_O_2_ to generate OH^•^ was facilitated by the redox pair Ce^3+^/Ce^4+^ to give complete removal of ciprofloxacin within 5 h, with a mineralisation rate of 77% in 3 h [146]. HKUST (metal organic framework)-derived Cu nanoparticles were embedded within a three-dimensional rGO network to give the E-Fenton catalyst [147]. It was proposed that the zero-valent Cu was oxidised to Cu^+^, which then catalysed the decomposition of the electrogenerated H_2_O_2_ to give OH^•^ and as a result the Cu^+^ was converted to Cu^2+^. The conducting catalyst layer, serving as the cathode, facilitated the reduction of Cu^2+^ to generate Cu^+^, leading to the efficient recycling of the Cu^+^/Cu^2+^ redox couple. Metal-free E-Fenton has also been recently proposed, as graphitic and pyridinic N sites on graphene-based materials appear to function as active sites for both the electrogeneration of H_2_O_2_ and the activation of OH^•^ radicals [54,126,148].

## 3. Conclusions and Future Perspectives

Graphene-based materials have attracted considerable interest, both from a fundamental viewpoint and in terms of their potential applications, and this family is one of the most studied, surpassing all other two-dimensional materials. It is no surprise that these materials are now finding applications in E-Fenton and, when combined with other carbon-based materials, they have the potential to give true iron- and metal-free E-Fenton catalysts. This would have a significant impact in terms of cost and environmental concerns. As shown earlier, rGO can be employed effectively in several ways, supported onto carbon or graphite electrodes or cloth, combined with CNTs, iron and other metal oxide catalysts, formed as aerogels and used in gas diffusion electrodes and doped with N and other elements, with N-doping appearing to be the best option in E-Fenton. It certainly appears that GO, with its very good conductivity when reduced to give rGO, high surface area, and good stability, will be used increasingly in future research in E-Fenton, leading to new and exciting developments. While the electrocatalytic generation of H_2_O_2_ is considered as an undesirable product in many research fields, such as batteries and fuel cells, tailoring the properties of the graphene-based composite with doping or by combining it with other materials to give a two-electron oxygen reduction reaction has significant potential not only in E-Fenton, but there are a number of other technologies, such as antimicrobial, medical, bleaching, gas scrubbing and refinery applications, that would benefit from the in situ generation of H_2_O_2_, eliminating the need for its storage.

In order for E-Fenton to emerge and be well integrated into real wastewater treatment facilities, new and more efficient Fenton catalysts are required. While heterogenous E-Fenton addresses many of the limitations of homogeneous E-Fenton, more efficient catalysts that are capable of generating high yields of H_2_O_2_, while catalysing the efficient conversion of H_2_O_2_ into OH^•^, reducing or eliminating the release of metal ions, such as Fe^3+^ or Fe^2+^, into the water, are required. These catalysts will also need to exhibit high stability, enabling their use over multiple E-Fenton cycles, with low energy demand. This will require the development of new Fenton catalysts and graphene composites have a clear role to play in these developments, most likely in terms of composites formed using other new and emerging materials, including other two-dimensional materials, and by doping.

In terms of possible new materials that could, in the future, be combined with graphene-based materials, MXenes deserve a special mention. These are exciting two-dimensional materials [149,150] that could be combined with graphene-based materials and possibly exploited in E-Fenton. Indeed, it has been shown that the MXenes have a high density of oxygen adsorption sites [151]. The further development of three-dimensional graphene composites is another interesting possibility. The three-dimensional framework gives high porosity, high surface area and facilitates ion diffusion, with numerous active sites that can enhance the rate of electron transfer. This approach could be further refined with the appropriate heteroatom doping of graphene composites. While N-doped graphene has been employed successfully in E-Fenton, dual or multiple dopants may exert even more beneficial effects. Halogen doping, F, Cl, Br and I, has been used to tailor the catalytic activity of carbon and graphene-based composites [152]. The charge accumulation at the doped halogens create a strong dipole [153] and this may enhance the adsorption of oxygen, making halogen doping interesting for E-Fenton applications. The recent use of gas diffusion electrodes is also important, as these electrodes have the potential to deliver much higher amounts of oxygen to the Fenton catalyst. It is very clear that the application of graphene-based materials in E-Fenton is still in its infancy and new developments will be seen in the next decade.

However, the application of graphene-based materials, as composites, or combined with other materials, and/or with doping, in E-Fenton has a number of challenges that must be overcome before significant advances are made. While remarkable progress has been made in the synthesis of graphene flakes, GO and rGO, a cost-effective large-scale synthesis is needed before these applications become a reality. The cost effective production of graphene and its oxides is required, but control over reproducibility and quality is equally important. Wet graphite exfoliation methods, including chemical and electrochemical processes, are promising in terms of scalability and cost, but concerns still remain over the reproducibility of these approaches and the quality of the final graphene product. Nevertheless, there is evidence to show that the oxygen reduction reaction is promoted at graphene edges, rather than basal planes, making these wet graphite exfoliation methods possibly suitable for the scaling up and production of graphene-based cathodes that can be employed in E-Fenton.

It is also difficult to precisely control the doping levels of graphene-based materials. At another level, there are some concerns in terms of the environmental impact of GO and its potential adverse effects on aquatic ecosystems [154]. In particular, GO contains polar oxygen-containing groups, making it more soluble in water. These environmental concerns need to be addressed by fabricating highly stable graphene-based cathodes that prevent the leaching of GO flakes into the environment. Secondly, cathodes containing a graphene-based catalyst will require regeneration or some suitable disposal to prevent secondary pollution. 

Nevertheless, with further advancements in the synthesis, scale-up and processing of graphene-based composites and electrodes, it is very likely that new Fenton catalysts and, indeed, metal-free Fenton catalysts can be fabricated, leading to innovations in advanced oxidation processes for the protection of water resources.

## Figures and Tables

**Table 1:** Summary of graphene-based felt electrodes in generating H_2_O_2_ or OH^•^ and in the removal of contaminants.

System	Pollutant	Experimental Conditions	*k*/min^−1^	H_2_O_2_/OH^•^	Ref.
Ferrocene-rGO/graphite	Cipro-Floxacin	V = 150 mL, E = −1.5 V, A = 10 cm^2^, t = 30 min, air sparging	0.035 (acidic) 0.222 (neutral)	OH^•^: 426 µM(acidic) 247 µM (neutral)	[58]
rGO-LCD	Cipro-Floxacin	V = 150 mL, E = −1.5 V, A = 10 cm^2^, t = 30 min, 1 L min^−1^ air flow	0.019 (neutral) 0.034 (acidic)	H_2_O_2_: 45 mg L^−1^ (acidic) 20 mg L^−1^ (neutral)	[73]
rGO-paste	Cipro-Floxacin	V = 400 mL, t = 45 min, E = −0.62 V, 1.2 L min^−1^ O_2_	0.0056 (acidic)	H_2_O_2_: 22 mg L^−1^ (acidic)	[72]
rGO ink/carbon	Phenol	V = 80 mL, t = 120 min, 0.2 L min^−1^ air flow, A = 6.3 cm^2^, I = 1.25 A cm^−2^	0.0157 (acidic)	H_2_O_2_: 2.81 mg L^−1^ cm^−2^	[71]
rGO/graphite cloth	Orange II Methy blue Sulfadiazine Phenol	V = 100 mL, A = 5.0 cm^2^, E = −0.9 V, t = 60 min	0.52 (acidic) 0.37 (acidic) 0.62 (acidic) 0.37 (acidic)	H_2_O_2_: 7.7 mg h^−1^ cm^−2^ (pH 7) 2.2 mg h^−1^ cm^−2^ (pH 5)	[40]
Flow-cell rGO	Sulfadiazine	Flow through system, 7 mL min^−1^, I = 50 mA	-	H_2_O_2_: 4.4 mg h^−1^ cm^−2^ (pH 7)	[3]
rGO/C felt	Imatinib	V = 150 mL, A = 12 cm^2^, air flow, I = 16.6 mA cm^−2^, t = 8 h	0.22 (acidic)	-	[55]
rGO dip coated/C felt	Cipro-floxacin Carba-mazepine	V = 300 mL, disc electrode 80 mm diameter, E = −1.5 V, t = 180 min.	0.37 (acidic) 0.20 (neutral) 0.35 (acidic) 0.08 (neutral)	H_2_O_2_: 175 mg L^−1^ (pH 7) 81 mg L^−1^ (pH 3)	[59]
rGO/C felt	Reactive Black 5	V = 250 mL, A = 82 cm^2^, E = −0.65 V, t = 180 min	-	H_2_O_2_: 0.26 mM	[57]
rGO C fibre Brush	Phenol	V = 250 mL, A = 46,665 cm^2^, I = 1.25 mA, t = 180 min	0.06 (acidic)	H_2_O_2_: 4.23 mg L^−1^ cm^−2^	[6]
Optimised graphite system				H_2_O_2_: 0.74 mg h^−1^ cm^−2^ 45 mg L^−1^	[76] [77]

**Table 2:** Summary of porosity and H_2_O_2_ generation rate for various porous graphene-based composites.

System	Surface Area/m^2^ g^−1^	Pore Diameter/nm	H_2_O_2_	Pollutant	Ref.
CNT/rGO	256.9	16.9	100 mg L^−1^	Methylene blue	[89]
3D rGO	280.15	7.34	-	EDTA-Ni	[87]
3D rGO foam	-	(100–600) × 10^3^	4.25 mg L^−1^ cm^−3^	Phenol	[90]
rGO/GDC	132	-	28.19 mg h^−1^ cm^−2^	Nalidixic acid	[101]
FeOOH aerogel	798–925	-	-	Sulfamethoxazole	[102]
rGO composite	459	3.9	85 mg L^−1^	Phthalic acid esters	[103]

**Table 3:** Leaching rates of iron from iron-containing graphene-based composites.

System	Iron Leaching	H_2_O_2_	OH^•^	Ref.
Fe/Cu/FeO_2_/rGO	2.0–3.1%	47.78 µM	-	[135]
Fe_3_O_4_/rGO	<1%, 0.02 mg L^−1^	-	177.2 µM	[65]
Fe_3_O_4_/rGO	2.4%	-	-	[136]
Fe_3_O_4_/rGO	0.02 mg L^−1^	-	-	[137]
Fe_3_O_4_/N-rGO, GDC	<9.5%, 0.009 mM	-	64 µM	[7]
Fe_2_O_3_/rGO aerogel	2.3 mg L^−1^	4.3 mg L^−1^	-	[88]
Fe_3_O_4_/CNT-rGO	<2 mg L^−1^	40 mg L^−1^	-	[85]
Fe_3_O_4_/N-rGO aerogel	0.33 mg L^−1^	-	-	[128]
